# Neoadjuvant Therapy for Localized Pancreatic Cancer

**DOI:** 10.3390/cancers18061011

**Published:** 2026-03-20

**Authors:** Jordan McKean, Patrick Underwood, Abdul Qahar K. Yasinzai, Thomas George, Ilyas Sahin, Jesus Fabregas, Bashar Qumseya, Aleksey Novikov, Tiffany Chua, Alessandro Paniccia, Kathryn E. Hitchcock, Steven Hughes, Ibrahim Nassour

**Affiliations:** 1 Department of Surgery, Division of Surgical Oncology, University of Florida, Gainesville, FL 32608, USA; jordan.mckean@surgery.ufl.edu (J.M.);; 2 Department of Medicine, Division of Hematology & Oncology, University of Florida, Gainesville, FL 32610, USAthom.george@medicine.ufl.edu (T.G.); 3 Massachusetts General Hospital Cancer Center, Harvard Medical School, Boston, MA 02114, USA; 4 Memorial Cancer Institute, Memorial Healthcare System, Hollywood, FL 33021, USA; jfabregas@mhs.net; 5 I-Health Institute, Florida Atlantic University, Boca Raton, FL 33431, USA; 6 Department of Medicine, Division of Gastroenterology, University of Florida, Gainesville, FL 32610, USA; 7 Department of Surgery, Division of Surgical Oncology, University of Pittsburgh Medical Center, Pittsburgh, PA 15213, USA; 8 Department of Radiation Oncology, University of Florida, Gainesville, FL 32610, USA

**Keywords:** neoadjuvant therapy, chemotherapy, pancreatic cancer

## Abstract

Background/Objectives: Pancreatic ductal adenocarcinoma is an aggressive malignancy with poor long-term survival. While surgical resection followed by adjuvant chemotherapy remains the standard of care for resectable disease, a substantial proportion of patients fail to receive postoperative therapy. Neoadjuvant treatment has emerged as an alternative strategy aimed at improving delivery of systemic therapy, increasing margin-negative resections, and better assessing tumor biology. This review summarizes major clinical trials evaluating neoadjuvant approaches in resectable and borderline resectable pancreatic cancer. Methods: A narrative review of published randomized and phase II–III clinical trials was performed, focusing on studies comparing neoadjuvant therapy with upfront surgery, trials comparing different neoadjuvant chemotherapy regimens, and studies evaluating the addition of neoadjuvant radiation therapy. Ongoing phase III trials were also reviewed. Results: Multiple trials demonstrated improved rates of chemotherapy delivery and higher R0 resection rates with neoadjuvant therapy. Some studies showed survival benefits (mOS ranging from 15.7 to 37 months), particularly in borderline resectable disease. While others failed to demonstrate an overall survival advantage compared with upfront surgery, especially in resectable disease. Trials comparing different neoadjuvant regimens and those evaluating the addition of radiation have yielded largely comparable outcomes, highlighting variability in treatment response and study design. Conclusions: Current evidence supports the feasibility and safety of neoadjuvant therapy in localized pancreatic cancer; however, survival benefits remain inconsistent, especially in resectable disease. Results underscore the need for well-powered randomized trials especially in Western populations, improved patient selection, and biologic stratification to better define the optimal role of neoadjuvant therapy.

## Simple Summary

Pancreatic cancer is one of the deadliest cancers, largely because it is often diagnosed late and treatment options are limited. Surgery offers the only chance for cure, but many patients are unable to receive chemotherapy after surgery due to complications or slow recovery. Giving chemotherapy before surgery, known as neoadjuvant therapy, has been proposed as a way to ensure more patients receive full treatment and to better select those who may benefit from surgery. This review summarizes major clinical trials that have studied neoadjuvant treatment for patients with localized pancreatic cancer. We discuss studies comparing chemotherapy before surgery to surgery first, trials comparing different chemotherapy regimens, and trials examining the addition of radiation. Overall, while neoadjuvant therapy shows promise, results remain mixed, and further large studies are needed to define which patients benefit most from this approach.

## 1. Introduction

Pancreatic ductal adenocarcinoma (PDAC) is the third-leading cause of cancer deaths in the United States and projected to be the second-leading cause of cancer deaths by the year 2030 [1]. The burden of this disease is largely owed to its high mortality rate with a reported 5-year survival rate of 13%. This is, in part, due to late stage at diagnosis with only 10–15% of individuals having operable disease leaving many individuals with advanced stages at diagnosis [1].

Treatment of non-metastatic PDAC focuses on multimodal therapy with surgical resection being the cornerstone and systemic therapy with or without radiation as an adjunct to improve oncologic outcomes. Several studies have shown a significant survival benefit with adjuvant chemotherapy [2,3,4,5]. Unfortunately, due to complications or prolonged recovery from surgery, it is estimated that 20–50% of patients do not receive adjuvant therapy. This has increased the interest in investigating the benefit of neoadjuvant therapy in patients with PDAC.

The management of resectable and borderline resectable pancreatic cancer is largely based on anatomy. A 2009 consensus statement issued by the American Hepato-Pancreato-Biliary Association in conjunction with the Society of Surgical Oncology and Society for Surgery of the Alimentary Tract recommends upfront surgical resection for resectable disease and neoadjuvant therapy for borderline resectable pancreatic cancer [6]. The current National Comprehensive Cancer Network (NCCN) guidelines have evolved and recommend upfront surgical resection or neoadjuvant therapy for resectable disease, while advocating neoadjuvant treatment for all cases of borderline resectable disease.

The rationale for neoadjuvant therapy in PDAC is threefold. First, it ensures a higher proportion of patients receive chemotherapy; since many patients never make it to treatment, delivering systemic therapy prior to surgery may better address potential micro-metastases. Second, it can downstage tumors therefore improving the likelihood of an R0 resection. Finally, it offers an opportunity to assess tumor biology upfront, identifying patients with unfavorable disease who may progress rapidly while on chemotherapy [7].

This review will discuss existing major randomized trials studying neoadjuvant therapy in patients with localized (resectable and borderline resectable) PDAC, including trials that compare neoadjuvant therapy to upfront resection, that compare different neoadjuvant regimen, and finally that study the addition of neoadjuvant radiation (Table 1).

## 2. Review of Major Clinical Trials Comparing Neoadjuvant Therapy to Upfront Resection

In 2019, the Study Group of Preoperative Therapy for Pancreatic Cancer (Prep) and Japanese Study Group of Adjuvant Therapy for Pancreatic Cancer (JSAP) reported a randomized phase II/III trial on neoadjuvant gemcitabine and S-1 followed by surgery then adjuvant S-1 versus upfront resection followed by adjuvant S-1 (Prep-02/JSAP05) [8]. Participants in the study had treatment naïve PDAC with resectable tumors. A total of 364 patients were enrolled in 57 centers (182 in the neoadjuvant group and 182 in the upfront resection group). The R0 resection was 93% in the neoadjuvant group and 82% in the upfront surgery group. The median survival was 37 months in the neoadjuvant therapy compared to 26.6 months (hazard ratio [HR] 0.73; 95% CI 0.56–0.95; *p* = 0.018). The 3-year overall survival was 50.2% and 36.6% respectively. The authors concluded that neoadjuvant therapy was superior to upfront resection in resectable pancreatic cancer.

In 2020, The PREOPANC-1 trial was published investigating perioperative chemoradiotherapy compared to immediate surgery for resectable and borderline resectable PDAC [9]. This was a randomized phase III trial that enrolled 248 patients from 16 centers; 120 in the neoadjuvant group, which consisted of three cycles of gemcitabine combined with radiotherapy during the second cycle and surgery followed by four cycles of Gemcitabine, and 128 in the immediate surgery group, which was followed by six cycles of Gemcitabine. The resection rate was 61% in the preoperative chemoradiation and 72% in the upfront resection group (*p* = 0.058). The R0 resection rate was 71% and 40% (*p* ≤ 0.001), respectively. The median overall survival in the preoperative chemoradiotherapy group was 16 months compared to 14.3 months in the immediate surgery group (HR 0.78; 95% CI 0.58–1.05; *p* = 0.096). A subgroup analysis showed no difference in overall survival between both groups in resectable disease but showed improved overall survival for borderline resectable disease treated with neoadjuvant therapy. The long term follow-up showed a median OS of 15.7 months in the neoadjuvant chemoradiotherapy group and 14.3 months in the upfront surgery group (HR 0.73; 95% CI 0.56 to 0.96; *p* = 0.025). Survival estimates at 5 years were 20.5% (95% CI, 14.2 to 29.8) for the neoadjuvant chemo-radiotherapy group and 6.5% (95% CI, 3.1 to 13.7) for the upfront surgery group [10]. The authors concluded that neoadjuvant therapy was superior to upfront resection in borderline and resectable pancreatic cancer.

In 2022, the PANACHE01-PRODIGE48 study was published and investigated the use of neoadjuvant therapy in resectable PDAC [11,12]. This was a non-comparative Phase II trial where 146 patients with resectable PDAC were randomized 2:2:1 to four cycles of neoadjuvant mFOLFIRINOX (*n* = 70) or FOLFOX (*n* = 50) or upfront resection (*n* = 26) in 28 centers. The resection rates were 74%, 68% and 81% respectively. The 1-year overall survival rates were 84.3%, 71.4% and 82.1%, respectively. The 1-year overall event-free survival rates were 51.4%, 43.1% and 38.7%, with corresponding median event-free survival of 12.4, 11 and 6.8 months, respectively. This study showed the feasibility and oncologic efficacy of perioperative mFOLFIRINOX.

In 2022, the NEONAX trial was published investigating perioperative or adjuvant-only gemcitabine plus nab-paclitaxel for resectable PDAC [13]. This was a randomized phase II trial that enrolled 118 patients in 22 German centers: 59 in the perioperative group (2 months before and 4 months after surgery) and 59 in the adjuvant group (6 months of therapy). The primary endpoint of the trial was DFS rate at 18 months in the so-called modified ITT population (mITT). The mITT comprised patients with R0/R1 PDAC resection having received at least one cycle of neoadjuvant chemotherapy and subsequent R0/R1 tumor resection in the perioperative arm and at least one cycle of adjuvant chemotherapy in the upfront surgery arm. The resection rate was 69% in the perioperative group and 78% in the upfront resection group. The R0 resection rate was 88% and 67%, respectively. The trial did not reach its primary endpoint, which was a DFS of 55% at 18 months in either arm. The results of the trial showed an 18-month DFS of 33.3% (95% CI 18.5% to 48.1%) in the perioperative group and 41.4% (95% CI 20.7% to 62.0%) in the upfront resection group. The median survival was 14.1 (95% CI 10.2–16.8 months) and 17 months (95% CI 10.9–25.1 months), respectively. Secondary analysis in the ITT population demonstrated an 18-month DFS of 30.8% (95% CI 18.3% to 43.2%) in the perioperative group versus 19.3% (95% CI 8.0% to 30.6%) in the upfront surgery group. Overall survival was 25.5 (95% CI 19.7–29.7 months) versus 16.7 months (95% CI 11.6–22.2 months), respectively. This study reinforced the feasibility and safety of neoadjuvant therapy in resectable PDAC.

In 2022, the ESPAC-5F trial was published and explored the role of different neoadjuvant therapies to include 2 months of gemcitabine plus capecitabine (GEMCAP) or 2 months of FOLFIRINOX or capecitabine-based chemoradiotherapy versus upfront surgery in borderline resectable disease in 90 patients [14]. This was a phase 2 feasibility randomized trial recruiting at 16 centers. The primary outcomes of interest were recruitment rate and R0 + R1 resection rate. When comparing any neoadjuvant treatment versus immediate surgery there was no difference in the primary outcome. The resection rate was 55% in the neoadjuvant group and 68% in the upfront resection group (*p* = 0.33). The R0 resection rate was 23% and 14% (*p* = 0.49), respectively. Secondary outcomes showed improved rate of 1-year overall survival in the generic neoadjuvant therapy group (76%) compared to the immediate surgery group (39%; HR 0.29, 95% CI 0.14–0.60, *p* = 0.0052). The 1-year overall survival was 78% (95% CI 60–100) for the neoadjuvant gemcitabine plus capecitabine group, 84% (95% CI 70–100) for the neoadjuvant FOLFIRINOX group, and 60% (95% CI 37–97) for the neoadjuvant chemoradiotherapy group (*p* = 0.0028). The authors concluded that short course (8-week) neoadjuvant therapy had a significant survival benefit compared with immediate surgery in borderline resectable PDAC.

In 2024, The NORPACT-1 trial was published and investigated 2 months of neoadjuvant FOLFIRINOX versus upfront surgery for resectable PDAC [15]. This was a phase 2 trial done in 12 centers and recruited 140 patients, 77 in the neoadjuvant group and 63 in the upfront surgery group. The resection rate was 82% in the neoadjuvant group and 89% in the upfront resection group (*p* = 0.24). The R0 resection rate was 56% and 39% (*p* = 0.018), respectively. The median overall survival was 25.1 months (95% CI 17.2–34.9) in the neoadjuvant group compared with 38.5 months (95% CI 27.6—not reached) in the upfront surgery group. The proportion of patients alive at 18 months by ITT was 60% (95% CI 49–71) in the neoadjuvant group and 73% (62–84) in the upfront surgery group. Notably, only 60% of patients completed four cycles of chemotherapy. This trial showed that four cycles of neoadjuvant therapy did not confer a survival benefit compared to upfront surgery.

Taken together, these studies reflect the growing momentum toward a neoadjuvant approach in both resectable and borderline resectable PDAC. Trials like Prep-02/JSAP05 demonstrated an impressive survival benefit and higher resection rates with neoadjuvant gemcitabine plus S-1 as compared with upfront surgery in resectable PDAC. Meanwhile, ESPAC-5F underscored improved 1-yr survival rates with neoadjuvant approaches in borderline resectable disease. However, other trials such as NORPACT-1 trial cautioned that neoadjuvant systemic chemotherapy might not always translate to better survival as compared to upfront surgery. Overall, these findings reinforce the heterogeneous results of neoadjuvant therapy in PDAC, emphasizing the need for additional well powered trials to identify the most effective regimens and refine treatment duration.

## 3. Review of Major Clinical Trials Comparing Different Neoadjuvant Therapies

In 2021, the SWOG S1505 was published, and it investigated 3 months of neoadjuvant mFOLFIRINOX versus Gemcitabine/nab-paclitaxel followed by surgery then adjuvant therapy with the same agent. It was a phase 2 randomized clinical trial with a pick the winner design that recruited 102 patients, 55 in the mFOLFIRINOX group and 47 in the Gemcitabine/nab-paclitaxel group. The study did not demonstrate an improved OS with perioperative chemotherapy, compared with historical data from adjuvant trials in resectable PDAC. The resection rate was 73% in the mFOLFIRINOX group and 70% in the Gemcitabine/nab-paclitaxel group and the R0 resection was 85% in both groups. The overall survival analysis revealed a median overall survival of 22.4 months for the mFOLFIRINOX group and 23.6 months in the gemcitabine/nab-Paclitaxel with a respective 2-year overall survival of 47% and 48% [16].

In 2023, The PREOPANC-2 trial was published as an abstract. It was a phase 3 randomized trial performed in 19 centers comparing the use of eight cycles of neoadjuvant FOLFIRINOX followed by surgery versus three cycles neoadjuvant gemcitabine with hypofractionated radiotherapy followed by surgery in borderline resectable and resectable PDAC [17]. In this study, only the gemcitabine with hypofractionated radiotherapy group utilized adjuvant treatment with four cycles of gemcitabine. It recruited 375 patients, 188 in the FOLFIRINOX group and 187 in the chemoradiation group. Resection rates were 77% in the FOLFIRINOX group and 75% in the chemoradiation group (*p* = 0.69). The R0 resection was 61% and 67% (*p* = 0.28), respectively. There was no difference in overall survival between the two treatment groups with the FOLFIRINOX group having a median overall survival of 21.9 months and the chemoradiation group having a median overall survival of 21.3 months (HR 0.87; 95% CI 0.68–1.12, *p* = 0.28).

In summary, SWOG S1505 demonstrated comparable resection and R0 rates between three months of neoadjuvant mFOLFIRINOX versus Gemcitabine/nab-paclitaxel in resectable PDAC, but neither arm showed a clear survival advantage. Meanwhile, PREOPANC-2 compared eight cycles of neoadjuvant FOLFIRINOX to three cycles of neoadjuvant gemcitabine with hypofractionated radiotherapy and again found no significant difference in resection rates, R0 resections or OS. Together these trials underscore the challenges in establishing a definitive perioperative regimen that confers a major OS advantage in resectable or borderline resectable PDAC.

## 4. Review of Major Clinical Trials Evaluating the Addition of Neoadjuvant Radiation

In 2022, the Alliance A021501 trial was published and investigated the role of adding neoadjuvant hypo-fractionated radiation to neoadjuvant mFOLFIRINOX (eight cycles in the chemotherapy group and seven cycles in the radiation group) for borderline resectable PDAC. This was a randomized phase 2 trial that recruited 126 patients. Among the first 30 evaluable patients enrolled to each arm, 57% in the chemotherapy group and 33% in the radiation group had undergone R0 resection, leading to closure of latter arm but continuation to full enrollment of the chemotherapy arm. Among patients who underwent pancreatectomy, R0 resection was achieved in 88% in the chemotherapy group and 74% in the radiation group. The 18-month OS rate was 66.7% (95% CI, 56.1–79.4%) and 47.3% (95% CI 35.8–62.5%) respectively. The median overall survival was 29.8 (95% CI, 21.1–36.6) months and 17.1 (95% CI, 12.8–24.4) months, respectively.

In 2024, the PANDAS/PRODIGE 44 was published as an abstract and investigated the role of conventional neoadjuvant chemoradiation in patients who received neoadjuvant mFOLFIRINOX for borderline resectable PDAC. This was a phase 2 randomized trial that included 110 patients who received four cycles of mFOLFIRINOX. Exactly 54 were randomized to two additional cycles of mFOLFIRINOX followed by surgery and adjuvant therapy, and 56 patients underwent chemoradiation (50.4 Gy in 28 fractions with capecitabine 5 days a week). The resection rates were 69% in the chemotherapy group and 55% in the chemoradiation group. The R0 resection rates were 54.1 and 58.1%, respectively. The median overall survival was 32.8 months (95% CI: 22.7–55.4) and 30 months (95% CI: 16.5-nr), respectively.

It has been challenging to define the role of radiation therapy in the neoadjuvant setting. Further studies are needed to understand which patient population may benefit from this local therapy.

## 5. Review of Ongoing Neoadjuvant Trials

The Alliance A021806 is a phase III study based in the United States and Canada evaluating perioperative mFOLFIRINOX (eight neoadjuvant, four adjuvant cycles) versus adjuvant (12 cycles) mFOLFIRINOX in patients with resectable PDAC. Primary outcome is overall survival, with secondary outcomes including resection rates and disease-free survival.

The PREOPANC-3 is a phase III study in Europe, examining perioperative (eight neoadjuvant, four adjuvant cycles) versus adjuvant (12 cycles) mFOLFIRINOX in patients with resectable PDAC. The primary outcome is overall survival, with secondary outcomes including resection rates and progression-free survival.

## 6. Discussion

Over the last decade, there has been a shift in the management of localized pancreatic cancer from upfront surgery followed by adjuvant therapy to neoadjuvant therapy [18]. This is the result of an increased understanding of the aggressive biology of pancreatic cancer which is thought to be a systemic disease at diagnosis and the realization that a large proportion of patients fail to receive multimodal therapy due to poor recovery from surgery [19]. Coupled with the success of neoadjuvant therapy in other GI malignancies, this approach has become more attractive. The advantages are multifold: neoadjuvant therapy increases the delivery of multimodal therapy as it ensures that a higher proportion of patients receive chemotherapy, has the potential to downstage the disease as evidenced by lower incidences of pN0 disease, is shown to increase R0 resection rates across multiple trials, and could treat micro metastatic disease selecting out patients who have bad biology and will ultimately have early recurrence.

A key rationale for neoadjuvant therapy in resectable pancreatic ductal adenocarcinoma is biological selection, allowing tumor behavior to declare itself before major surgery. Dynamic changes in CA19-9 during treatment provide a practical surrogate of systemic disease control: a substantial decline or normalization is generally associated with improved survival and higher likelihood of durable benefit from resection, whereas persistently elevated or rising levels suggest chemoresistance or occult metastatic disease. Similarly, early radiographic or clinical progression on chemotherapy identifies patients with aggressive biology who are unlikely to benefit from upfront surgery, thereby sparing them operative morbidity without meaningful survival gain. Emerging data on circulating tumor DNA (ctDNA) further refine this concept, as detectable or persistent ctDNA during or after neoadjuvant therapy may indicate minimal residual disease and higher relapse risk, while ctDNA clearance could reflect effective systemic control. Together, these markers support a shift from purely anatomic to biologically informed decision-making in resectable pancreatic cancer.

Despite the perceived advantages of neoadjuvant, there is no definitive large phase 3 randomized trial, especially in resectable pancreatic cancer, to solidify this approach in the Western world. On the other hand, there are multiple phase 3 randomized trials proving the success of adjuvant therapy with median overall survival up to 54.4 months with mFOLFIRINOX [2]. This is partially the result of a Darwinian selection of patients that had favorable biology with low CA19-9, survived surgical resection and were able to start adjuvant therapy. Yet there is still reluctance in complete adoption of neoadjuvant approach for all resectable PDAC patients. Possible disadvantages of neoadjuvant therapy are the potential complications associated with biliary stents or the necessity for biliary decompression during treatment, the occasional need of multiple biopsies to prove cancer, functional status deterioration during systemic therapy and the delay of surgery. These events could lead to disease progression and failure to proceed to the ultimate goal which is surgical resection.

The trials presented above, especially the ones that utilize contemporary systemic therapy, are small phase II studies that do not conclusively provide evidence to support the use of a neoadjuvant approach, with some even questioning it. It is true that there is increase in delivery of multimodal therapy and increase in R0 resection, but this did not consistently translate into an obvious improved overall survival. The main challenge of some these studies is the use of short course neoadjuvant systemic therapy (2 months), the use of the toxic FOLFIRINOX regimen instead of the modified version leading to decreased overall delivery, the lack of stratification by biological marker and just relying on anatomic staging and finally not being able to change regimen when there is no radiological or biological response.

Until the publication of the results of The Alliance A021806 and the PREOPANC-3 trials, there will be an equipoise between neoadjuvant therapy and upfront resection in resectable PDAC. Both trials will provide Level I evidence for timing of systemic therapy. These are among the first adequately powered phase III trials to compare perioperative versus adjuvant-only mFOLFIRINOX in resectable PDAC. A positive trial could establish *neoadjuvant therapy plus surgery* as the new standard, while a null result would reinforce the current surgery-first paradigm. By focusing on overall survival, these trials will determine whether earlier systemic treatment truly improves long-term outcomes, rather than just surrogate outcomes like resection rates or pathological response. If perioperative therapy demonstrates superior survival, guideline panels (e.g., NCCN, ASCO, ESMO) could move from permissive recommendations toward strong endorsement of neoadjuvant regimens in appropriately selected resectable PDAC patients. Conversely, if the adjuvant approach remains superior, recommendations will lean toward surgery first, with optimized postoperative therapy. Data from these trials will reduce geographic and institutional variability in how clinicians approach resectable pancreatic cancer—a setting where practice currently varies widely because of the absence of definitive evidence

Beyond the debate over treatment sequencing, significant unmet needs persist in improving early detection tools, optimizing systemic therapy to enhance overall outcomes, and leveraging biological markers to enable more individualized treatment strategies for this aggressive disease.

## 7. Conclusions

Current evidence supports the feasibility and safety of neoadjuvant therapy in localized pancreatic cancer; however, survival benefits remain inconsistent, especially in resectable disease. Results underscore the need for well-powered randomized trials especially in Western populations, improved patient selection, and biologic stratification to better define the optimal role of neoadjuvant therapy.

## Figures and Tables

**Table 1:** Abbreviations: CXRT, chemoradiotherapy; GEMCAP, gemcitabine/capecitabine; ITT, intention to treat; mDFS, median disease-free survival; mITT, modified intention to treat; mOS, median overall survival; RT, radiotherapy.

Country	Treatment Arms	Trial Phase Tumor Stage	N (Arm 1)	N (Arm 2)	N (Arm 3)	N (Arm 4)	Primary Endpoint	Secondary Endpoint	Resection Rates	R0 Resection Rate
Japan	Arm 1: Preoperative gemcitabine + S-1 Arm 2: Upfront surgery + adjuvant S-1	II/III Resectable	182	182	-	-	mOS Arm 1: 37.0 mo Arm 2: 26.6 mo	mDFS Arm 1: 14.3 mo Arm 2: 11.3 mo	Arm 1: 93% Arm 2: 91.1%	Arm 1: 85.9% Arm 2: 82%
Netherlands	Arm 1: Preoperative gemcitabine + RT Arm 2: Upfront surgery + adjuvant	III Resectable Borderline	120	128	-	-	mOS (mITT) Arm 1: 15.7 mo Arm 2: 14.3 mo	mDFS Arm 1: 8.1 mo Arm 2: 7.7 mo	Arm 1: 61% Arm 2: 72%	Arm 1: 71% Arm 2: 40%
France	Arm 1: Neoadjuvant mFOLFIRNOX Arm 2: Neoadjuvant FOLFOX Arm 3: Upfront surgery	II Resectable	72	50	28	-	1-year OS Arm 1: 84.3% Arm 2: 71.4% Arm 3: 82.1%	1-year DFS Arm 1: 51.4% Arm 2: 43.1% Arm 3: 38.7%	Arm 1: 68% Arm 2: 75% Arm 3: 81%	Arm 1: 74% Arm 2: 74.5% Arm 3: 55.6%
Germany	Arm 1: Preoperative gemcitabine/nab-paclitaxel Arm 2: Upfront surgery + adjuvant gemcitabine/nab-paclitaxel	II Resectable	59	59	-	-	18 mo DFS (ITT) Arm 1: 30.8% Arm 2: 19.3%	mOS Arm 1: 25.5 mo Arm 2: 16.7 mo	Arm 1: 69% Arm 2: 78%	Arm 1: 88% Arm 2: 67%
United Kingdom	Arm 1: Upfront surgery Arm 2: Preoperative GEMCAP Arm 3: Preoperative FOLFIRINOX Arm 4: Preoperative CXRT	II Borderline	32	20	20	16	Recruitment Rate: 21 patients per year	1-year OS Arm 1: 39% Arms 2–4 (neoadjuvant group): 76%	Arm 1: 68% Arms 2–4 (neoadjuvant group): 55%	Arm 1: 14% Arms 2–4 (neoadjuvant group): 23%
International	Arm 1: Preoperative FOLFIRINOX + adjuvant mFOLFIRINOX Arm 2: Adjuvant mFOLFIRINOX	II Resectable	77	63	-	-	18 mo OS Arm 1: 60% Arm 2: 73%	mOS Arm 1: 25.1 mo Arm 2: 38.5 mo	Arm 1: 82% Arm 2: 89%	Arm 1: 56% Arm 2: 39%
United States	Arm 1: Preoperative mFOLFIRINOX Arm 2: Preoperative gemcitabine/paclitaxel	II Resectable	55	47	-	-	2-year OS Arm 1: 47% Arm 2: 48%	mOS Arm 1: 22.4 mo Arm 2: 23.6 mo	Arm 1: 73% Arm 2: 70%	Arm 1: 85% Arm 2: 85%
Netherlands	Arm 1: Preoperative mFOLFIRINOX Arm 2: Preoperative gemcitabine + RT	II/III Resectable Borderline	375	187	-	-	mOS Arm 1: 21.9 mo Arm 2: 21.3 mo	Adverse Events Arm 1: 49% Arm 2: 43%	Arm 1: 77% Arm 2: 75%	Arm 1: 61% Arm 2: 67%

## Data Availability

No new data were created or analyzed in this study.
